# Evaluation of Antibacterial Activity of Some Traditionally Used Medicinal Plants against Human Pathogenic Bacteria

**DOI:** 10.1155/2015/265425

**Published:** 2015-02-09

**Authors:** Bishnu P. Marasini, Pankaj Baral, Pratibha Aryal, Kashi R. Ghimire, Sanjiv Neupane, Nabaraj Dahal, Anjana Singh, Laxman Ghimire, Kanti Shrestha

**Affiliations:** ^1^Central Department of Microbiology, Tribhuvan University, Kirtipur, Kathmandu, Nepal; ^2^Nepal Academy of Science and Technology (NAST), Khumaltar, Lalitpur, Nepal; ^3^Department of Pathobiological Science, School of Veterinary Medicine, Louisiana State University, Baton Rouge, LA 70803, USA; ^4^Department of Biochemistry, Faculty of Medicine Siriraj Hospital, Mahidol University, Bangkok 10700, Thailand; ^5^Patan Hospital, Patan, Lalitpur, Nepal

## Abstract

The worldwide increase of multidrug resistance in both community- and health-care associated bacterial infections has impaired the current antimicrobial therapy, warranting the search for other alternatives. We aimed to find the *in vitro* antibacterial activity of ethanolic extracts of 16 different traditionally used medicinal plants of Nepal against 13 clinical and 2 reference bacterial species using microbroth dilution method. The evaluated plants species were found to exert a range of *in vitro* growth inhibitory action against the tested bacterial species, and *Cynodon dactylon* was found to exhibit moderate inhibitory action against 13 bacterial species including methicillin-resistant *Staphylococcus aureus*, imipenem-resistant *Pseudomonas aeruginosa*, multidrug-resistant *Salmonella typhi*, and *S. typhimurium*. The minimum inhibitory concentration (MIC) values of tested ethanolic extracts were found from 31 to >25,000 *μ*g/mL. Notably, ethanolic extracts of *Cinnamomum camphora, Curculigo orchioides*, and *Curcuma longa* exhibited the highest antibacterial activity against *S. pyogenes* with a MIC of 49, 49, and 195 *μ*g/mL, respectively; whereas chloroform fraction of *Cynodon dactylon* exhibited best antibacterial activity against *S. aureus* with a MIC of 31 *μ*g/mL. Among all, *C. dactylon, C. camphora, C. orchioides*, and *C. longa* plant extracts displayed a potential antibacterial activity of MIC < 100 *μ*g/mL.

## 1. Introduction

The emergence and spread of multidrug-resistant (MDR) bacterial pathogens have substantially threatened the current antibacterial therapy [1]. MDR bacterial infections often lead to increased mortality, longer length of stays in hospitals, and higher cost of treatment and care [1, 2]. The most problematic bacteria include, but are not limited to, extended-spectrum *β*-lactamase-producing* Escherichia coli* (ESBL-EC) and* Klebsiella pneumoniae* (ESBL-KP), carbapenem-resistant* Enterobacteriaceae*,* Pseudomonas aeruginosa*, and* Acinetobacter baumannii, *hospital-acquired methicillin-resistant* Staphylococcus aureus* (MRSA), and vancomycin resistant* Enterococcus* (VRE) [2–4]. Thus, Infectious Diseases Society of America has recognized MRSA, VRE, ESBL-EP, ESBL-KP, and* Pseudomonas aeruginosa* as notorious pathogens among the six major pathogens to which therapies with effective newer antimicrobials are urgently required [1, 4].

The therapeutic options for these pathogens are extremely limited and physicians are forced to use expensive or previously discarded drugs, such as colistin, that are associated with significant side effect to the patients' health [1]. Therefore, it is necessary to search the other alternatives that can potentially be effective in the treatment of these problematic bacterial infections. The usefulness of plant extracts for antimicrobial therapy and/or other diseases have been observed to be promising remedies since ancient time in Chinese medicine, Ayurveda, Arabic, and Unani medicine [5]. The inclusion of traditionally used medicines including phytomedicine, if they prove safe and effective, into national health care system is suggested by World Health Organization [5]. Although a large number of medicinal plants (>500 plants) have been reported to be used by Nepalese people since a long time for primary health care, there has been a paucity in data regarding their* in vitro* or* in vivo* efficacy [6]. In this study, we aimed to determine the* in vitro* antibacterial activity of extracts from some selected medicinal plants from Nepal against the most common bacterial pathogens including MDR bacteria. The sixteen selected plants in this study have remained as integral part of traditional medicine in Nepal to treat different types of infectious diseases, including diarrhea, respiratory tract infection, cholera, and skin and wound infections (Table 1).

## 2. Materials and Methods

### 2.1. Plant Materials and Extract Preparation

Popular plants used in traditional medicine by the Nepalese people across the country, based upon previous ethnobotanical literatures and potential medicinal values as judged by local healers, were screened and selected to include in the present study. The collected plants were identified at the National Herbarium and Plant Laboratories, Kathmandu, and the voucher specimens have been deposited at National Academy of Science and Technology, Nepal. The most potential parts of the plants that could exhibit antimicrobial activity as judged by the traditional trend for the parts to be used for the treatment of diseases were selected for the study (Table 1). All collected plant materials were air-dried at room temperature under shade, and pulverized into fine powder and processed for extract isolation. The elaborated steps for the isolation and processing of the plant materials are covered in supplementary information (details are in Supplementary Material available online at http://dx.doi.org/10.1155/2015/265425).

### 2.2. Antimicrobial Testing

#### 2.2.1. Bacteria

A total of 15 bacterial species were used in the study, which includes ten Gram-negative bacteria (clinical strains: ESBL-EC,* Citrobacter freundii*,* Enterobacter cloacae*, ESBL-KP, imipenem-resistant* P. aeruginosa*,* S. enteritidis*, MDR* S. typhi*,* S. typhimurium,* and* Vibrio cholerae* and a reference strain* E. coli* ATCC 25922) and five Gram-positive bacteria (clinical strains: MRSA,* Enterococcus faecalis*,* Streptococcus agalactiae,* and* S. pyogenes* and a reference stain* S. aureus* ATCC 25923). All of the tested bacteria were identified and characterized by culturing in the specific appropriate media followed by the rapid testing (Gram's stain, catalase, oxidase, coagulase, and bile solubility) and the biochemical testing (IMViC (Indole, methyl red, Voges-Proskauer, and citrate), TSI (triple sugar iron), O/F (oxidation/fermentation), urease, and nitrate reduction). Antimicrobial susceptibility was performed for all tested clinical bacterial strains by disk diffusion method following Clinical and Laboratory Standards Institute (CLSI) recommendations [7]. For the characterization of MDR bacteria, a recently standardized definition for MDR by European Centre for Disease Prevention and Control (ECDC) and Centers for Disease Control and Prevention (CDC) was followed; accordingly MDR was defined as acquired nonsusceptibility to at least one agent in three or more antimicrobial categories [3]. The antibiotic resistance pattern of tested bacteria is shown in Supplemental Table 1.

### 2.3. Standardization of Bacterial Suspension

The bacterial suspensions were standardized following the CLSI guidelines for aerobic bacteria [8]. All of the tested bacteria were grown in Mueller Hinton Broth (MHB, Hi-Media) for 18–24 h, followed by the matching of bacterial suspension to the turbidity equivalent to 0.5 McFarland solution (1-2 × 10^8^ CFU/mL) with the addition of sterile saline.

### 2.4. Minimum Inhibitory Concentration (MIC) Determination

Antibacterial activities of the extracts were first screened by agar-well diffusion method as described previously [9]. The MIC testing was performed for all the plant extracts that were judged as active (inhibition zone > 7 mm) against at least one test organism by agar-well diffusion method. The MIC values were determined by microbroth dilution method using 96-well plates (detailed procedure is in Supplementary Material).

## 3. Results and Discussion

A total of 16 ethanolic extracts from 16 nonrepetitive plants were tested for antibacterial activity qualitatively as well as quantitatively against the 13 clinical bacterial species and the 2 reference bacterial species. The MDR and non-MDR clinical strains used in this work were isolated from human infections diagnosed in a tertiary care hospital of Nepal (Supplemental Table 1). Although some extracts exhibited a good antibacterial activity towards different tested bacterial isolates, many plant extracts exhibited a limited antibacterial activity against the test bacterial isolates as judged by their higher MIC values (Table 2). However, these extracts showed the larger inhibition zone (by agar-well diffusion method, Supplemental Table 2) as well as low MIC values (Table 2) against the Gram-positive bacteria when compared against the Gram-negative bacteria. Our results are in agreement with several previous findings demonstrating greater activity of the plant extracts towards Gram-positive bacteria compared to Gram-negative bacteria [10, 11]. One of the most plausible reasons behind such observation, as also mentioned by others, is the different nature of cell wall among Gram-positive and Gram-negative bacteria [11]; however efflux pump system of Gram-negative bacteria may mediate for such difference [12].

The strongest antibacterial activity (MIC = 49 *μ*g/mL) was observed for the extracts from* Curculigo orchioides* and* Cinnamomum camphora *against* S. pyogenes,* followed by* Curcuma longa* extract against* E. faecalis* (MIC = 98 *μ*g/mL) (Table 2), establishing the traditional values of the use of these plants for the remedies of bronchitis, and skin and/or wound infections.* C. dactylon* extract showed a broad-spectrum antibacterial activity compared to other extracts inhibiting 12 bacterial species including MDR-ST, ESBL-EC, ESBL-KP, and MRSA with MIC values ranging from 391 to 3125 *μ*g/mL (Table 2). Therefore, ethanolic extractof* C. dactylon* fractionated into different solvents to evaluate its antibacterial property and chloroform fraction exhibited good inhibitory effect with MIC values ranging from 31 to 250 *μ*g/mL (Table 3). The minimum bactericidal concentration (MBC) values of the plant extracts against tested bacterial species were also found largely higher and ranged from 98 to >25,000 *μ*g/mL (Supplemental Table 3). The observed MIC and MBC values of the plant extracts in this study are in a range or lower than the values of other plant extracts reported from different countries [13–15], but are higher than those values that have been reported in a number of reports [11, 16, 17]. The high temperature during soxhlet extraction in our study might be responsible for the degradation of antibacterial active ingredients.

In our study, the plant extracts from* C. longa*,* G. biloba,* and* R. serpentina* were demonstrated to inhibit the growth of all tested Gram-positive bacteria, whereas only the plant extract from* C. dactylon* was observed to inhibit the growth of all tested Gram-negative bacteria except* C. freundii* (Table 2 and Supplemental Table 2). Recent studies on antimicrobial effect of* C. dactylon* had shown that it is also effective against some other bacteria, such as* Bacillus subtilis* and* Aeromonas hydrophila *[18]. Although certain number of extracts exhibited good antibacterial potency, in contrary to our expectation, a limited antibacterial potency of some plants suggest that there is no complete agreement between the traditional use of medicinal plants in the crude form for the remedy of infectious diseases. Further study, however, is still warranted to explore their effectiveness to inhibit the growth of parasites, viruses, and/or fungi. Another possibility for the limited antibacterial potency of some plants may be due to soxhlet extraction method and use of crude extracts. Instead of it, percolation extraction, subfraction, semipure compound, or pure compounds isolated from these plants might exhibit better antibacterial activity. Although we have shown the potent* in vitro* activity of few traditional plant extracts (e.g.,* C. camphora, C. orchioides, and C. longa*) for certain bacteria, we are not certain about if such activity will be translated* in vivo. *Future epidemiological studies are necessary to understand the effectiveness/impact of use of extracts from such medicinal plants in population.

## 4. Conclusion

In this study we evaluated the antibacterial activity of 16 commonly used traditional medicinal plants from Nepal. Some extracts displayed a potent antibacterial activity with MIC <100 *μ*g/mL, indicating that these plants could be a good source for the antibacterials to combat MDR bacterial infections. Further studies are necessary for these potent plant extracts to evaluate the other parameters of antimicrobial efficacy (e.g.,* in vivo* efficacy, toxicity, and antimycobacterial, antiviral, and antiparasitic activity).

## Supplementary Material

The elaborated steps for the isolation and processing of the plant materials are covered in Supplementary Material. The antibiotic resistance pattern of tested bacteria is shown in Supplemental Table 1. The MIC values were determined by microbroth dilution method using 96-well plates (detailed procedure is in Supplementary Material). The MDR and non-MDR clinical strains used in this work were isolated from human infections diagnosed in a tertiary care hospital of Nepal (Supplemental Table 1). However, these extracts showed the larger inhibition zone (by agar-well diffusion method, Supplemental Table 2). The minimum bactericidal concentration (MBC) values of the plant extracts against tested bacterial species were found to be largely higher and ranged from 98 to >25,000 𝜇g/mL (Supplemental Table 3). In our study, the plant extracts from *C. longa*, *G. biloba*, and *R. serpentina* were demonstrated to inhibit the growth of all tested Gram-positive bacteria, whereas only the plant extract from *C. dactylon* was observed to inhibit the growth of all tested Gram-negative bacteria except *C. freundii* (Supplemental Table 2).

## Figures and Tables

**Table 1:** Ethnomedicinal data of selected plants of Nepal used for the antibacterial activity evaluation.

Voucher number	Botanical name (family)	Common (local) name	Parts used traditionally	Ethnomedicinal uses	References
KS-7	*Acorus calamus* L. (Araceae)	Sweet flag, Calamus (Bojho)	Rhizomes	Cough, respiratory tract infections, skin disease, toothache, dysentery	[19, 20]
KS-8	*Adhatoda vasica* L. (Acanthaceae)	Malabar nut (Asuro)	Leaves	Bronchitis, asthma, diarrhea, dysentery, as anthelmintic	[21, 22]
KS-9	*Artemisia vulgaris* L. (Compositae)	Mugwort (Titepati)	Aerial parts	Antiseptic, diarrhea, dysmenorrhea, asthma, as anthelmintic	[21, 22]
KS-10	*Asparagus racemosus *Wild. (Liliaceae)	Shatavari (Kurilo)	Rhizomes, stem	Urinary troubles, diarrhea, as antidysenteric,	[19, 21, 22]
KS-11	*Centella asiatica* L. (Umbelliferae)	Water Pennywort (Ghodtapre)	Whole plant	Skin diseases, urinary tract infection, leprosy, ulcers, indigestion	[19, 21, 22]
KS-12	*Cinnamomum camphora* L. (Lauraceae)	Camphor (Kapoor)	Leaves, seeds, bark	As antiseptic, bronchitis, bronchopneumonia, epilepsy	[21, 22]
KS-13	*Curculigo orchioides* Gaertn. (Amaryllidaceae)	Black musale (Kalo Musali, Dhusilo)	Rhizomes	Diarrhea, dysentery, demulcent, diuretic, skin disease	[21, 22]
KS-14	*Curcuma longa* L. (Zingiberaceae)	Turmeric (Besar, Haldi)	Rhizomes	Antiseptic, cuts, wounds, as anthelmintic, jaundice, liver disorders	[19, 21, 22]
KS-15	*Cuscuta reflexa* Roxb. (Cuscutaceae)	Dodder (Aakase beli)	Whole plant	Fever, stomachache, rheumatism, cuts, wounds, as purgative, as anthelmintic	[19, 21, 22]
KS-16	*Cynodon dactylon *L. (Poaceae)	Bermudagrass, Doob grass (Dubo)	Whole plant	Cuts, wounds, indigestion, genitourinary disorders	[19, 21, 22]
KS-17	*Drymaria cordata* Wild. (Caryophyllaceae)	Whitesnow, Chickweed (Abijalo)	Whole plant	Laxative, as antifebrile, as antisinusitis	[19, 21, 22]
KS-18	*Eupatorium adenophorum* Spreng. (Compositae)	Crofton weed, Eupatory (Banmara)	Leaves	Cuts, wounds, boils, as antiseptic	[21, 22]
KS-19	*Ginkgo biloba* Spreng. (Ginkgoaceae)	Ginkgo (Ginkgo)	Leaves	As antiaging, used to treat Alzheimer's disease, as anticoldness, as antinumbness	[21, 22]
KS-20	*Psidium guajava* L. (Myrtaceae)	Guava (Amba, Belauti)	Leaves, bark	Diarrhea, dysentery, cuts, wounds, piles, cholera	[21, 22]
KS-21	*Rauwolfia serpentina *L. (Apocynaceae)	Serpentine (Sarpagandha)	Root	As antidysenteric, as antidote to snakebite, cuts, wounds, and boils	[19, 21, 22]
KS-22	*Swertia chirayita* Roxb. (Gentianaceae)	Chiretta (Chiraito)	Aerial parts	Skin disease, eczema, as anthelmintic, as antidiarrheal, dyspepsia	[21, 22]

**Table 2:** Minimum inhibitory concentration (MIC, *μ*g/mL) of ethanol plant extracts and reference antibiotics against bacteria.

Plant species	Gram-negative bacteria										Gram-positive bacteria				
	ESBL-EC	*Ec *	*Vc *	*Cf *	MDR-ST	*Ecl *	IRPA	*Stm *	*Se *	ESBL-KP	MRSA	*Ef *	*Sa *	*Sp *	*Sal *
*Acorus calamus *	—	—	3125	—	12500	—	6250	—	—	—	—	6250	—	3125	6250
*Adhatoda vasica *	—	6250	—	—	—	—	—	—	—	—	—	—	—	12500	12500
*Artemisia vulgaris *	—	—	3125	—	6250	—	6250	6250	—	6250	3125	6250	3125	391	—
*Asparagus racemosus *	—	—	—	—	12500	—	6250	—	—	—	—	12500	—	391	—
*Centella asiatica *	—	—	—	—	—	—	—	—	—	—	12500	—	6250	3125	12500
*Cinnamomum camphora *	—	6250	6250	—	—	—	—	781	—	3125	—	3125	3125	**49**	—
*Curculigo orchioides *	—	—	12500	—	—	6250	12500	—	—	6250	12500	—	12500	**49**	—
*Curcuma longa *	—	6250	1562	—	—	—	—	—	—	—	6250	98	3125	195	6250
*Cuscuta reflexa *	—	12500	1562	—	6250	12500	6250	—	—	6250	12500	12500	6250	781	—
*Cynodon dactylon *	1562	781	781	—	1562	6250	781	1562	6250	391	391	—	195	781	3125
*Drymaria cordata *	—	—	6250	—	—	12500	6250	6250	—	6250	12500	—	6250	3125	12500
*Eupatorium adenophorum *	—	12500	12500	—	6250	—	3125	—	—	6250	3125	3125	3125	6250	—
*Ginkgo biloba *	—	—	—	—	—	—	—	—	—	—	12500	12500	6250	6250	12500
*Psidium guajava *	—	—	12500	3125	6250	6250	781	3125	6250	3125	6250	3125	6250	3125	—
*Rauwolfia serpentina *	—	12500	—	12500	—	6250	3125	6250	—	6250	6250	6250	6250	3125	3125
*Swertia chirayita *	—	12500	12500	—	—	3125	—	—	—	12500	6250	—	6250	—	—
Gentamicin^a^	64	<1	<1	32	<1	16	64	<1	<1	64	—	—	—	—	—
Vancomicin^b^	—	—	—	—	—	—	—	—	—	—	<1	<1	<1	<1	<1

“—”: not tested because plant extracts did not show the inhibitory effect by agar-well diffusion method or not suitable to test (for reference drug); *Cf*: *Citrobacter freundii*; *Ecl*: *Enterobacter cloacae*; *Ef*: *Enterococcus faecalis*, *Ec*: *Escherichia coli *(25922); ESBL-EC: extended-spectrum *β*-lactamase-producing *Escherichia coli*; ESBL-KP*:*extended-spectrum *β*-lactamase-producing*-Klebsiella pneumoniae*; IRPA: imipenem-resistant* Pseudomonas aeruginosa, Sa*: *Staphylococcus aureus* (ATCC 25923); MRSA: methicillin-resistant *Staphylococcus aureus*; *Sal*: *Streptococcus agalactiae*; *Se*: *Salmonella enteritidis*; *Sp*: *Streptococcus pyogenes*; MDR*-*ST: multidrug-resistant* Salmonella typhi*; *Stm*: *Salmonella typhimurium*; *Vc*: *Vibrio cholera*e. Gentamicin and vancomycin were used as reference drugs for Gram-negative bacteria and Gram-positive bacteria, respectively.

a and b = Reference drugs used for the Gram-negative and Gram-positive bacteria, respectively.

**Table 3:** MIC (*μ*g/mL) of subfraction of ethanol extracts of *Cynodon dactylon* and reference antibiotics against bacteria.

Solvent system	Gram-negative bacteria										Gram-positive bacteria				
	ESBL-EC	*Ec *	*Vc *	*Cf *	MDR-ST	*Ecl *	IRPA	*Stm *	*Se *	ESBL-KP	MRSA	*Ef *	*Sa *	*Sp *	*Sal *
n-Hexane	—	—	—	—	—	—	—	500	—	—	—	—	—	—	—
Chloroform	250	63	—	—	—	125	—	500	—	—	63	—	31	—	—
n-Butanol	—	—	—	—	—	—	—	—	—	250	125	500	63	—	—
Water	—	—	500	—	—	—	—	—	—	—	—	—	—	—	—
Gentamicin^a^	64	<1	<1	32	<1	16	64	<1	<1	64	—	—	—	—	—
Vancomicin^b^	—	—	—	—	—	—	—	—	—	—	<1	<1	<1	<1	<1

“—”: not tested because plant extracts did not show the inhibitory effect by agar-well diffusion method or not suitable to test (for reference drug); *Cf*: *Citrobacter freundii*; *Ecl*: *Enterobacter cloacae*; *Ef*: *Enterococcus faecalis*; *Ec*: *Escherichia coli *(25922); ESBL-EC: extended-spectrum *β*-lactamase-producing *Escherichia coli*; ESBL-KP: extended-spectrum *β*-lactamase-producing*-Klebsiella pneumoniae*; IRPA: imipenem-resistant* Pseudomonas aeruginosa; Sa*: *Staphylococcus aureus* (ATCC 25923); MRSA: methicillin-resistant *Staphylococcus aureus*; *Sal*: *Streptococcus agalactiae*; *Se*: *Salmonella enteritidis*; *Sp*: *Streptococcus pyogenes*; MDR*-*ST: multidrug-resistant* Salmonella typhi*; *Stm*: *Salmonella typhimurium*; *Vc*: *Vibrio cholera*e; a and b: gentamicin and vancomycin were used as reference drugs for Gram-negative bacteria and Gram-positive bacteria, respectively.
