# Supplementary material for: Evaluation of Antibacterial Activity of Some Traditionally Used Medicinal Plants against Human Pathogenic Bacteria
Source: Biomed Res Int. 2015 Feb 9;2015:265425. doi: 10.1155/2015/265425 (PMC4337259; doi:10.1155/2015/265425)
Supplement: Supplementary file 1 — The elaborated steps for the isolation and processing of the plant materials are covered in Supplementary Material. The antibiotic resistance pattern of tested bacteria is shown in Supplemental Table 1. The MIC values were determined by microbroth dilution method using 96-well plates (detailed procedure is in Supplementary Material). The MDR and non-MDR clinical strains used in this work were isolated from human infections diagnosed in a tertiary care hospital of Nepal (Supplemental Table 1). However, these extracts showed the larger inhibition zone (by agar-well diffusion method, Supplemental Table 2). The minimum bactericidal concentration (MBC) values of the plant extracts against tested bacterial species were found to be largely higher and ranged from 98 to >25,000 𝜇g/mL (Supplemental Table 3). In our study, the plant extracts from C. longa, G. biloba, and R. serpentina were demonstrated to inhibit the growth of all tested Gram-positive bacteria, whereas only the plant extract from C. dactylon was observed to inhibit the growth of all tested Gram-negative bacteria except C. freundii (Supplemental Table 2). [file 265425.f1.pdf]

Supplementary data

1.1 Materials and methods

Isolation of plant extract

One hundred grams of plant powder was subjected to Soxhlet extraction after mixing with 80% ethanol (250 ml) for 6-12 h. The mixture was then filtered and the same process was repeated thrice. The filtrates were combined and the solvent in the mixture was then removed by using rotary vacuum evaporator at 60°C until a thick dry residue was formed. Extract of *C. dactylon* was dissolved in water, n-hexane was mixed and shaken well, and separated by separating funnel. The process was repeated with chloroform and n-butanol. The yields of different plant materials were measured. The residue was first dissolved in 10% (v/v) dimethyl sulphoxide (DMSO) in Tris buffer saline (TBS) at pH 7.6 to make a final concentration of 1 gm/mL of stock solution and then diluted with TBS if needed.

Agar-well diffusion testing

Antibacterial activities of the plant extracts investigated were first evaluated by agar-well diffusion method. The standardized cultures of test bacteria were first evenly spread onto the surface of Mueller Hinton Agar (MHA; HiMedia) plates using sterile cotton swabs. For the *Streptococcus* species, MHA plates containing 5% sheep blood were used. Three wells (6 mm diameter) were made in each plate with sterile cork borer. Fifty microliters of each of the plant extract (50000 µg/mL) was added in one well of each plate. In the remaining two wells, 50 µl of 5% DMSO (as negative control) with TBS and 50 µl of reference antibiotic solution (as positive control) were added. Gentamicin (200 µg/mL) and vancomycin (1 µg/mL) were used as reference antibiotic for Gram-negative bacteria and Gram-positive bacteria, respectively. Diffusion of extracts, antibiotics and DMSO were allowed at room temperature for 1 h. All of the plates were then covered with lids and incubated at 37°C for 24 h. After incubation, plates were observed for zone of bacterial growth inhibition. The size of inhibition zones was measured and antimicrobial activity of the plant extracts was expressed in

terms of the average diameter of inhibition zone in millimeters. Those plant extracts that were unable to exhibit inhibition zone (inhibition zone diameter less than 7 mm) were considered non-active. Plant extracts demonstrating inhibition zone diameters between 7 and 10 mm were considered as moderately active and interpreted by "+", whereas other active plant extracts were interpreted by "++" and "+++" if the inhibition zone diameters were between 10 and 15 mm and >15 mm respectively. Each plant extract was tested in triplicate with two independent experiments and mean values of inhibition zone diameters were taken.

Determination of minimum inhibitory concentration and minimum bactericidal concentration

The minimum inhibitory concentration (MIC) values of the plant extracts were determined by microbroth dilution method. The test bacteria from the stock cultures were inoculated in MHB and incubated at 37°C under stirring for 24 h. The bacterial suspensions were then diluted with fresh MHB to achieve the turbidity equivalent to 0.5 McFarland standard. Different dilutions to get the final concentration ranging from 49 to 25000 μg/mL for the plant extract and 1 to 256 μg/mL for the reference antibiotics (gentamicin and vancomycin) were prepared in MHB directly in the wells of 96-well plates in a final volume of 200 μL. In each of these dilutions, 100 μL was of bacterial suspension (approx. 1.5 x 10^6 CFU/mL). The highest percentage of DMSO being used in the wells was 2.5 % and was not found to inhibit the growth of test bacteria. The wells containing 100 μL MHB, 100 μL bacterial inoculum and DMSO at a final concentration of 2.5 % served as negative control. The plates were covered with sterile plate sealer and were agitated to mix the contents of the wells by using shaker. Then the plates were incubated at 37°C for 18 h. After incubation, 40 μL of 0.2 mg/ml *p*-iodonitrotetrazolium violet indicator solution was added to every well in order to assess the bacterial growth. This indicator solution changes its color from colorless to red in the presence of bacterial growth and the degree of redness is a good indicator of inhibitory effect of extract or antibiotic on bacterial growth. After addition of this indicator solution, the plate was incubated for an additional 30 minutes. The MIC was defined as the lowest concentration of extract or antibiotic in which there was no visible growth of a test bacterium. To determine the MBC values, inoculations of the plant extracts with different concentrations

from the wells without indicator were inoculated onto MHA. The MBC was determined as the lowest concentration of extract that showed the complete inhibition of the bacterial growth. All the values of MIC and MBC are expressed as mean values of duplicate samples of three different experiments.

1.2 Results

Supplemental Table 1: Selected bacterial pathogens for testing the antimicrobial activity of plant extracts

**Bacteria**	**Type of Infection/disease caused**	**No. of antibiotic resistance**	**Susceptibility to**																
			Amoxicillin	Ampicillin	Cefixime	Cefotaxime	Ceftriaxone	Cepalexin	Chloramphenicol	Ciprofloxacin	Co-trimoxazole	Erythromycin	Methicillin	Nalidixic acid	Norfloxacin	Ofloxacin	Penicillin G	Tetracycline	Vancomycin
**Gram-negative pathogens**																			
ESBL-EC	UTI	10	R	R	R	R	R	R	S	-	S	-	-	R	R	R	-	R	-
Citrobacter freundii	Wound infection	7	R	S	R	R	S	R	R	-	R	-	-	S	S	S	-	R	-
Enterobacter cloacae	Wound infection	7	R	R	R	S	R	R	R	-	R	-	-	S	-	S	-	-	-
ESBL-KP	Blood-stream infection	8	R	R	R	R	R	-	R	R	S	-	-	S	S	S	R	S	-
IRPA	Wound infection	10	R	R		R	R	-	R	S	R	R	-	R	-	S	R	R	-
Salmonella enteritidis	Diarrhea	4	S	R	R	S	S	-	R	-	R	-	-	-	-	S	-	S	-
MDR-ST	Typhoid fever	3	S	R	S	S	S	-	R	-	R	-	-	-	-	S	-	S	-
Salmonella typhimurium	Diarrhea	2	S	R	S	S	S	-	S	-	S	-	-	-	-	S	-	R	-
Vibrio cholerae	Cholera	2	S	R	S	S	S	S	S	S	R	-	-	-	-	-	-	S	-
**Gram-positive pathogens**																			
MRSA	Wound infection	9	-	R	-	R	-	R	R	R	R	R	R	-	-	-	R	S	S
Enterococcus faecalis	UTI	2	-	R	-	-	-	S	S	S	S	S	S	-	-	-	R	S	-
S. agalactiae	Pneumonia	3	-	R	-	-	-	R	S	S	S	S	-	-	-	-	R	-	-
Streptococcus pyogenes	URTI	2	-	R	-	-	-	S	S	S	S	S	-	-	-	-	R	S	-

ESBL-EC, extended-spectrum β-lactamase producing *Escherichia coli;* ESBL-KP, extended-spectrum β-lactamase producing *Klebsiella pneumoniae* IRPA, imipenem-resistant *Pseudomonas aeruginosa;* MDR-ST, multidrug-resistant *Salmonella typhi* ; MRSA, methicillin-resistant *Staphylococcus aureus;* R = resistant, S = sensitive; RTI-upper respiratory tract infection, '-', not tested

Supplemental Table 2: Antibacterial activities of ethanol extracts of 16 medicinal plants by agar-well diffusion method

Plant species	Parts Used	Extract yield (%)	Gram–negative bacteria										Gram–positive bacteria				
			ESBL–EC	*Ec*	*Vc*	*Cf*	MDR-ST	*Ecl*	IRPA	*Stm*	*Se*	ESBL-KP	MRSA	*Ef*	*Sa*	*Sp*	*Sal*
Acorus calamus	Rhizomes	20.2	–	–	+	–	+	–	+	–	–	++	–	+++	–	++	++
Adhatoda vesica	Leaves	23.4	–	–	+	–	–	–	–	–	–	–	–	–	–	++	++
Artemisia vulgaris	AP	28.1	–	+	++	–	+	–	+	+	–	+	++	+	++	+++	–
Asparagus racemosus	Roots	16.2	–	–	–	–	+	–	+	–	–	–	–	+	–	+	–
Centella asiatica	Whole	30.9	–	–	–	–	–	–	–	–	–	–	++	–	++	++	++
Cinnamomum camphora	Leaves	21.8	–	+	+	–	–	–	–	++	–	++	–	++	+	+	–
Curculigo orchioides	Rhizomes	6.8	–	–	+	–	–	+	+	–	–	++	+	–	+	++	–
Curcuma longa	Rhizomes	30.8	–	+	+	–	–	–	–	–	–	–	++	+++	+	++	++
Cuscuta reflexa	WP	30.1	–	+	++	–	+	+	+	–	–	+	++	+	++	++	–
Cynodon dactylon	WP	15.2	++	++	++	–	++	++	++	+++	+	++	+++	–	+++	++	++
Drymaria cordata	WP	29.7	–	–	+	–	–	+	++	++	–	++	+	–	++	++	+
Eupatorium adenophorum	Leaves	26.9	–	+	++	–	+	–	+	–	–	+	++	+	++	+	–
Ginkgo biloba	Leaves	19.6	–	–	–	–	–	–	–	–	–	–	+	+	+	+	+
Psidium guajava	Leaves	17.5	–	–	+	+	+	+	+++	++	+	++	+++	++	++	+	–
Rauwolfia serpentina	Roots	16.4	–	+	–	+	–	+	++	++	–	++	++	++	+	++	++
Swertia chirayita	AP	9.6	–	+	+	–	–	+	–	–	–	+	++	–	++	–	–
Gentamicin[b]			+	++	+++	+	+++	++	+	+++	+++	+	nt	nt	nt	nt	nt
Vancomycin[c]			nt	nt	nt	nt	nt	nt	nt	nt	nt	Nt	+++	+++	+++	+++	+++

A zone of inhibition diameter ≥ 7 mm was considered positive, "+" = inhibition between 7 to 9 mm, "++" = inhibition between 10 to 14 mm, "+++" = inhibition >15 mm, "–" = no inhibition, nt= not tested; AP, aerial parts; WP, whole plant; *Cf = Citrobacter freundii; Ecl = Enterobacter cloacae; Ef = Enterococcus faecalis, Ec = Escherichia coli* (25922), ESBL-EC=extended-spectrum β-lactamase-producing *Escherichia coli*, ESBL-KP = extended-spectrum β-lactamase-producing -*Klebsiella pneumoniae*, IR *Pa* =imipenem- resistant *Pseudomonas aeruginosa, Sa = Staphylococcus aureus* (ATCC 25923), MRSA =methicillin-resistant *Staphylococcus aureus*, *Sal = Streptococcus agalactiae*, *Se = Salmonella enteritidis; Sp = Streptococcus pyogenes*, MDR-ST = multidrug-resistant *Salmonella typhi*, *Stm = Salmonella typhimurium*, *Vc = Vibrio cholerae*
Gentamicin and vancomycin were used as reference drugs for Gram-negative bacteria and Gram–positive bacteria, respectively

Supplemental Table 3: **Minimum bactericidal concentration (MBC, µg/mL) of ethanol plant extracts and reference antibiotics against bacteria**

Plant species	Gram–negative bacteria										Gram–positive bacteria				
	ESBL–EC	*Ec*	*Vc*	*Cf*	MDR-ST	*Ecl*	IRPA	*Stm*	*Se*	ESBL-KP	MRSA	*Ef*	*Sa*	*Sp*	*Sal*
Acorus calamus	–	–	6250	–	25000	–	25000	–	–	–	–	12500	–	6250	12500
Adhatoda vesica	–	25000	–	–	–	–	–	–	–	–	–	–	–	25000	25000
Artemisia vulgaris	–	–	6250	–	12500	–	25000	25000	–	25000	6250	25000	6250	1562	–
Asparagus racemosus	–	–	–	–	25000	–	>25000	–	–	–	–	25000	–	1562	–
Centella asiatica	–	–	–	–	–	–	–	–	–	–	12500	–	12500	12500	25000
Cinnamomum camphora	–	25000	25000	–	–	–	–	6250	–	6250	–	6250	25000	**98**	–
Curculigo orchioides	–	–	25000	–	–	25000	>25000	–	–	12500	25000	–	25000	**98**	–
Curcuma longa	–	25000	6250	–	–	–	–	–	–	–	25000	195	12500	781	25000
Cuscuta reflexa	–	12500	3125	–	12500	25000	25000	–	–	12500	25000	25000	12500	3125	–
Cynodon dactylon	6250	6250	3125	–	6250	25000	6250	3125	12500	6250	3125	–	1562	3125	6250
Drymaria cordata	–	–	25000	–	–	>25000	12500	12500	–	12500	25000	–	6250	6250	25000
Eupatorium adenophorum	–	>25000	25000	–	>25000	–	12500	–	–	25000	12500	25000	6250	25000	–
Ginkgo biloba	–	–	–	–	–	–	–	–	–	–	25000	25000	12500	12500	25000
Psidium guajava	–	–	25000	12500	25000	>25000	3125	12500	25000	12500	25000	12500	25000	12500	–
Rauwolfia serpentina	–	25000	–	25000	–	25000	6250	12500	–	12500	12500	12500	12500	6250	6250
Swertia chirayita	–	>25000	25000	–	–	6250	–	–	–	>25000	12500	–	12500	–	–
Gentamycin[b]	128	<1	<1	64	<1	32	128	<1	<1	64		–	–	–	–
Vancomycin[c]	–	–	–	–	–	–	–	–	–	–	<1	<1	<1	<1	<1

"–" = not tested because plant extracts did not show the inhibitory effect by agar-well diffusion method or not suitable to test (for reference drug), *Cf = Citrobacter freundii; Ecl = Enterobacter cloacae; Ef = Enterococcus faecalis, Ec = Escherichia coli* (25922), ESBL-EC=extended-spectrum β-lactamase-producing *Escherichia coli*, ESBL-KP = extended-spectrum β-lactamase-producing-*Klebsiella pneumoniae*, IRPA =imipenem-resistant *Pseudomonas aeruginosa, Sa = Staphylococcus aureus* (ATCC 25923), MRSA =methicillin-resistant *Staphylococcus aureus, Sal = Streptococcus agalactiae, Se = Salmonella enteritidis; Sp = Streptococcus pyogenes,* MDR-ST = multidrug-resistant *Salmonella typhi, Stm = Salmonella typhimurium, Vc = Vibrio cholera*e, Gentamicin and vancomycin were used as reference drugs for Gram-negative bacteria and Gram–positive bacteria, respectively.
